# Mixed-methods library evaluation integrating the patron, library, and external perspectives: The case of Namibia regional libraries

**DOI:** 10.1016/j.evalprogplan.2020.101782

**Published:** 2020-04

**Authors:** Moonjung Yim, Michelle Fellows, Chris Coward

**Affiliations:** a University of Washington Information School, Mary Gates Hall, Suite 095, Box 352840, Seattle, WA 98195-2840, USA; b Technology & Social Change Group, University of Washington Information School, Bloedel Hall 060, Seattle, WA 98195, USA

**Keywords:** Public library, Performance evaluation, Mixed-methods approach

## Abstract

The article presents a mixed-methods evaluation of regional libraries in Namibia, which incorporates three perspectives: the patron perspective (library users), the library perspective (library staff, management, and related officials), and the external perspective (including evaluators and monitoring data). Seven data collection methods were used: patron surveys, patron panel studies, focus group discussions, key informant and staff interviews, secondary data analysis, media analysis, and observations. The goal of the evaluation was to assess library performance for both formative and summative purposes by addressing evaluation questions on areas such as library services, use, and operations. Building upon the literature review of how mixed-methods approaches can contribute to library evaluation, the aim of this article is to show how a mixed-methods evaluation can be designed to examine multi-faceted library performance and to illustrate how the evaluation design allows information complementarity and can be utilized to present diverse viewpoints of the above three perspectives. The evaluation design, analysis process, and lessons learned from this study may be useful to evaluators engaged in evaluation of public services or programs (including public libraries) that examine multiple aspects of service performance and involve a variety of stakeholders.

## Highlights

• We present a mixed-methods evaluation of regional libraries in Namibia, which incorporates the patron, library, and external perspectives.
• We describe how data collection methods representing different perspectives were used and how the data were integrated.
• The article illustrates how a mixed-methods evaluation can be designed to examine multi-faceted library performance.
• The evaluation design also allows providing complementary information of different stakeholders’ views.
• The evaluation design, analysis process, and lessons learned from this study may be useful to evaluators engaged in evaluation of public services or programs that examine multiple aspects of service performance and involve a variety of stakeholders.

## 1 Introduction

The most basic purpose of libraries has been to provide people with access to information through the provision of books and other resources. However, the function of libraries can be further expanded when we perceive them as part of a social system: “they exist to serve the needs of people, to help them live, learn and develop and to act as part of the social glue which holds communities together” (Brophy, 2006, pp. 3). To fulfill their function to meet people’s information needs, libraries offer *services*—“the ways in which the library makes its resources available to users”—and *programs*—"[a]n activity or event (or series of events) scheduled by a library for the benefit of its patrons” (Gross, Mediavilla, & Walter, 2016, pp. 2 citing Reitz, 2014). These are provided within a certain *space* of facilities, collections, and equipment where patrons (library users) may pursue activities to gain information, either individually or through interactions with other patrons or library staff. Therefore, a library can be understood as a system of itself (Hernon, Altman, & Dugan, 2015), where its units, personnel, and patrons interact to meet the information needs of patrons.

Evaluating libraries and information services is ultimately about assessing “the effects of what they do” (Brophy, 2006, pp. 54). In this sense, evaluating library performance and impact are closely related. Library performance evaluation can help its stakeholders to: understand the relevance of services and efficiency of resource use, compare across similar organizations, assess against guidelines or standards, or justify and make decisions to sustain service provision (Brophy, 2006). Despite the recognition that library performance evaluation plays a significant role as a tool to guide management practices and fulfill accountability, there have been problems: a lack of consensus on the list and definition of performance measures, key decision makers not fully recognizing the value and utility of performance measures, and an absence of organizational structure in actively utilizing performance measures (Matthews, 2018). The challenges essentially stem from the complex context that library evaluation addresses, encompassing societal, administrative, technological, and professional (librarianship) aspects (Wallace, 2001). Moreover, library evaluation involves multiple stakeholders—such as library users, librarians, staff, government officials, and community members in the area where the library is located—and their views and values may vary (Brophy, 2006). Furthermore, indicators of operation, performance and impact—which will be further explained in Section 2.1.—involve both tangible and intangible factors which may not be easy to measure or assess. Especially for intangibles, there is a lack of consensus on a definitive assessment model, which exacerbates the complexity of library evaluation (White, 2007).

Given these challenges, this study examines the application of a mixed-methods approach in public library evaluation and illustrates how mixed-methods approaches in evaluation are critical to measuring library performance. More specifically, the aim will be twofold: (1) to show how a mixed-methods approach to library evaluation can be designed to assess multi-faceted library performance and (2) to explain with a case how the evaluation design allows for “information complementarity” and can be utilized to present diverse viewpoints (da Costa, Pegado, Ávila, & Coelho, 2013) of three categories of perspectives: the *patron perspective* (i.e. library users), the *library perspective* (i.e. library staff, management, and related officials), and the *external perspective* (i.e. others, including observers from the evaluation team, the media, and monitoring data). The evaluation design and analysis approach presented in this paper to assess areas such as effectiveness, use, service quality, and user satisfaction can help inform evaluators with the type of approach they can use for future library performance evaluation.

In this paper, we will first review previous works on library evaluation and how the application of mixed methods can contribute to the field. After introducing the case of Namibia’s regional libraries, we will explain the rationale underlying the performance evaluation’s design—with a closer look at each data collection method—and analysis procedure. Afterwards, we will present key findings which show how a mixed-methods approach was helpful to understanding library performance—especially in terms of resources provided, usage, and operations.^2^ We will revisit the potential of mixed methods in library evaluation, assess the study’s limitations, and end with discussions on lessons learned in evaluation procedure and methodology, providing implications for this project’s final analysis and future practice.

For this paper, the descriptions and explanations for evaluation design and analysis build upon an evaluation design report (Coward, Fellows, & Gordon, 2014), a data collection plan (TASCHA, 2017), and the findings of interim evaluation report (Coward, Fellows, Koepke, Rothschild, & Yim, 2019) of the Namibia regional libraries performance evaluation project.

## 2 Library evaluation

### 2.1 Models and aspects of library evaluation

One of the most frequently cited and widely utilized models for evaluating library performance is the logic model, otherwise referred to as Input-Process-Output-Outcomes(-Impact) model (Brophy, 2006; Hernon et al., 2015; Markless & Streatfield, 2013; Matthews, 2018; Orr, 1973; Poll & Payne, 2006). The model explains how a library is equipped with resources (input) which provide the capability to offer services (process); here, capability is utilized by library patrons through the use of information and services (output), potentially resulting in discernable changes in or consequences for patrons (outcomes) and may generate long-term effects (impact) (Brophy, 2006; Matthews, 2018 citing Orr, 1973; Poll & Payne, 2006). Yet, Markless and Streatfield (2013, pp. 24–25) argue that the performance evaluation logic model has limitations when applied to library service, in terms of establishing a clear connection between aggregated outputs on one hand and outcome and impact on the other: “No amount of monitoring of book loans…will tell you whether the items borrowed were actually read, let alone whether the targeted users were in any way affected by what they read, or whether they learnt anything.” This leads to the task of conceptualizing the links among input, process, output, outcomes, and impact elements in library performance context.

Many scholars have explored ways to conceptualize library performance, particularly through the examination of performance criteria such as economy, efficiency, effectiveness, productivity, service quality, use, and user satisfaction (Baker & Lancaster, 1991; Brophy, 2006; Hernon & Dugan, 2002; Hernon et al., 2015; Markless & Streatfield, 2013; Matthews, 2018). Among the criteria above, effectiveness, use, service quality, and user satisfaction are the areas most closely associated with findings presented in this study.^3^ *Effectiveness* addresses if the right products were provided, in alignment with fundamental aims (Brophy, 2006). *Use* indicates how patrons interact with the library (Hernon et al., 2015). *Service quality* entails aspects of “what” (deliverables) and “how” (the interaction between the library or service provider and its intended users) and is formed from users’ impression of service (Hernon & Dugan, 2002; Hernon et al., 2015; Matthews, 2018, pp. 278). In comparison, *satisfaction* is an emotional sense of contentment or discontentment, and in the library context, it has been said to have two components: *service encounter satisfaction* (“the degree of satisfaction or dissatisfaction experienced…in a specific service transaction”) and *overall service satisfaction* (“the level of client satisfaction or dissatisfaction based on multiple transactions or experiences”) (Hernon et al., 2015; Matthews, 2018, pp. 278).

### 2.2 Potential of mixed-methods approaches in library evaluation

Based on 19 different definitions of mixed-methods research from prominent scholars in the field (including Pat Bazeley, Huey Chen, John Creswell, Jennifer Greene, Donna Mertens, Margarete Sandelowski, Michael Q. Patton), Johnson, Onwuegbuzie, and Turner (2007, pp. 123) derived that mixed-methods research “combines elements of qualitative and quantitative research approaches (e.g. use of qualitative and quantitative viewpoints, data collection, analysis, inference techniques) for the broad purposes of breadth and depth of understanding and corroboration.” Bazeley (2018, pp. 7) specifies that “integration of data and analyses occur prior to drawing final conclusions about the topic of the investigation” in mixed-methods research. The highlight is placed on “integration” based on “purposeful interdependence between the different sources, methods, or approaches used” to “[reach] a common theoretical or research goal” (Bazeley, 2018, pp. 7–8).

Mixed-methods approaches support the evaluation of library performance by allowing for a comprehensive analysis of how parts and the whole of the organization deliver resources, services, and programs to meet the information needs of intended audiences—from the varying perspectives of multiple stakeholders—through the combination of qualitative and quantitative methods and approaches (Bloch et al., 2014; Greene, Benjamin, & Goodyear, 2001). Also, triangulation through mixed methods supports validation of collected data, assessment of findings’ consistency, and expansion of breadth and depth of analysis (BetterEvaluation, 2014).

Moreover, mixed-methods approaches offset the possible respective weaknesses of a solely quantitative or qualitative approach, as mixed methods can provide in-depth knowledge of the evaluation setting with considerations of contextual and cultural dimensions (otherwise a weakness of quantitative-only approaches) along with some degree of generalization of results and identification of group tendency (otherwise a weakness of qualitative-only approaches) (Creswell & Plano Clark, 2011; da Costa et al., 2013; Gosselin, Valiquette-Tessier, Vandette, & Romano, 2015). Achieving the balance is particularly important in library evaluation—most importantly, evaluating factors including input, process, output, outcome, and impact and aspects such as effectiveness, use, and user satisfaction cannot be done solely by either relying on figures and trends (from quantitative methods) or capturing contextual details (from qualitative methods). One might place higher priority on qualitative methods in library evaluation based on the notion that a library’s essential function is to serve local communities, and that understanding the socio-cultural context of library use (and non-use) is essential to gauging a library’s effectiveness. However, since public libraries are accountable to multiple stakeholders (often including local governments and taxpayers), it is also critical to find generalizable trends about library users (e.g. demographics) and their experiences (e.g. usage patterns). Moreover, generalizable quantitative data can be used to make comparisons across libraries, whether for national benchmarking efforts or understanding trends in library use and services at any scale.

Also, evaluation questions surrounding library performance—such as the questions of this study listed in Table 1 below—may be geared toward gaining “practical understandings” about the current status of library’s resources and services vis-à-vis patron needs and to inform action for future operations, rather than “validating the nature of reality, getting at the essence of some phenomenon, generating grounded theory, or deconstructing social constructions” (Patton, 2015, pp. 152). In such cases, the library evaluation aligns with pragmatism, and in turn with using mixed methods, because “pragmatism opens the door to multiple methods…as well as different forms of data collection and analysis”, without being “committed to any one system of philosophy and reality.” (Creswell, 2009, pp. 10–11)

**Table 1:** Evaluation questions (EQs) of phases 1, 2, and 3 of Namibia regional libraries performance evaluation project.

Phase 1	- EQ1: Was the MCC investment implemented according to plan?
Phases 2 and 3	- EQ2: What types of resources and programming are the libraries providing?^a^ - EQ3: Who uses the libraries and what do they do?^a^ - EQ4: Do students, job seekers and business people report outcomes such as improved test scores, job seeking and acquisition, and business creation and enhancement as a result of using the resources provided by the libraries? - EQ5: How sustainable are the libraries? - EQ6: How active is leadership in promoting and achieving the vision of the libraries? - EQ7: What is the influence of the libraries beyond their walls?

a EQs of primary interest in interim evaluation report.

There have been a number of studies on evaluating public library performance, mostly through the examination of use and/or outcomes (e.g. Antell (2004), Bhatt (2010), Dent and Goodman (2015), Gichohi, Onyancha, and Dulle (2017), Nielsen and Borlund (2011), Vakkari and Serola (2012), Vakkari et al. (2016)). However, few illustrate (1) an application of a mixed-methods approach and (2) an assessment reflecting multiple perspectives (i.e. among library service provider, user, etc.), while also providing (3) practical considerations for evaluators based on the lessons learned. Moreover, there are very few studies which examine in-depth how mixed-methods approaches can contribute to assessing multi-faceted library performance. This study attempts to bridge this gap. The evaluation design, analysis process, and lessons learned from this study may be useful to evaluators engaged in evaluation of public services or programs (including public libraries) that examine multiple aspects of service performance and involve a variety of stakeholders.

## 3 Namibia regional libraries

In late 2014, the Government of Namibia opened three newly constructed regional libraries in the towns of Oshakati (Oshana Region), Helao Nafidi (Ohangwena Region), and Gobabis (Omaheke Region). The libraries were designed to address community information needs throughout the region via provision of locally relevant programs, information services, study facilities, and information and communication technology (ICT) access, and to stimulate education and economic activities (TASCHA, n.d.; Millennium Challenge Corporation (MCC), 2016). The locations were chosen based on local need—considering factors like high population density, high poverty level, a limited presence of libraries in the region, and/or low secondary school performance (Coward et al., 2014)—and were created with support from the Millennium Challenge Corporation (MCC), a U.S. foreign aid agency providing grants to promote economic growth, reduce poverty, and support institutions (MCC, n.d.).

Each regional library provides access to ICT facilities and services, such as public access computers, basic computer training classes, WiFi access, and equipment for printing, scanning, and photocopying. The libraries also have study rooms, meeting rooms, conference halls, and sections that target children and job-seekers/waged employees/entrepreneurs. To provide services in more remote areas of the region, the libraries were launched with accessory mobile library units—large vehicles equipped with books and information technology (IT), including services that could be used outside of the vehicle (e.g. WiFi and a television screen). During the presidential speech at the opening ceremony of the library in Ohangwena, the important role of libraries in development was highlighted in terms of providing access to information to communities and promoting education, culture, and lifelong learning (Republic of Namibia, 2014). Also, the use of the libraries by young people and entrepreneurs was strongly encouraged to advance reading skills and economic opportunities (Republic of Namibia, 2014).

## 4 Evaluation design and analysis

### 4.1 Overview

The evaluation team was contracted to design and implement a performance evaluation of the regional libraries in the Oshana, Ohangwena, and Omaheke Regions. The evaluation was intended to serve two major stakeholders, MCC and the Namibia Library and Archives Service (NLAS)—for the former, the evaluation provided a summative assessment of effectiveness, efficiency, and sustainability of the libraries to inform future education related projects, while also performing an accountability function for citizens of Namibia and the United States; for the latter, the evaluation provided a formative assessment for improving the program in the three initial library locations and future libraries to be established throughout Namibia, in addition to the summative aspect (TASCHA, 2017).

We have seen in Section 2.1 that the logic model is one of the most widely utilized models for library performance evaluation. This evaluation also had a program logic model which can be summarized as below (Millennium Challenge Corporation (MCC), 2016):

• Inputs: Technical assistance and funding from MCC; existing library and administrative resources; effort of implementing organizations
• Outputs: Completed and opened regional libraries (including mobile library units)
• Immediate outcomes: Staffed libraries; implementation of programs; people visit to meet information and other (e.g. business, educational, or recreational) needs
• Intermediate outcomes: Business owners, job seekers improve business skills or find jobs; children obtain school subject knowledge and develop skills; educators improve their teaching skills; community members become more informed and empowered
• Ultimate impacts: Improved business activities and environment; community members become more educated and their literacy rates improve; enhanced state of well-being

The program logic model has a conceptual role in the evaluation. For instance, when we describe if or how a service is being used by patrons (output), we understand usage is shaped by what the library does to provide that service (process), and library activities depend on resources available (inputs). The evaluation^4^ also touches on the value of that service in terms of effects on patrons, like having improved access to information such as books or study materials online, or saving money in applying for jobs by utilizing library resources (early/immediate outcomes; as we will see in Section 5.1). The program logic was foundational to our research design (including evaluation questions, listed in Table 1) and interpretation of the data. As a model, it helps clarify evaluators’ thinking—to make sure we understand how the library might achieve the outcomes it is aiming for.

Data collection for the regional library performance evaluation involved three phases. The first phase focused on the process of planning, constructing, and equipping the libraries before they opened, and the findings were informed by key informant interviews, field observations, and a review of project documents conducted in 2015 (Sey & Fellows, 2016). Data collection for the second (2017) and third phases (2018) were mostly similar in terms of the methods and instruments used. The main evaluation questions covered in each phase are presented in Table 1.

This paper focuses on the process and findings from the *interim evaluation report* and is based on *phase two data collection*. The final evaluation report is planned for publication, following phase three data collection and analysis—which are out of scope of this article. For the interim evaluation report, it was decided to focus specifically on assessing service provision, operations, and use (EQ2 and EQ3) and reserve the subjects of outcomes, sustainability, leadership, and broader influence of the regional libraries (EQ4, EQ5, EQ6, EQ7) for the final evaluation report, as these areas were expected to take more time to manifest. Accordingly, it was hoped that findings from the interim evaluation report would illuminate issues the libraries could learn about and (conceivably) address before the end of the evaluation.

The evaluation project takes a mixed-methods approach. For data collection phases two and three, qualitative methods were designed “to understand the historical path the [libraries have] taken; current and on-going operational practices; user behaviors and opinions; and the implications for the effectiveness and sustainability of [the libraries]” (TASCHA, 2017, pp. 5–6). Quantitative methods were designed to provide statistical figures on in-depth characteristics of user subgroups and trends in usage and outcomes (TASCHA, 2017).

Patrons were grouped into three large categories based on evaluation priorities: learners and students (with an emphasis on youth learners), business section patrons, and other patrons (Coward et al., 2019):

• Learners and students^5^ : Comprised of respondents who answered “learner” or “student” as their primary occupation. This category can be further divided into “youth learners” (ages 15–19) and “adult learners and students” (ages 20 and above).
• Business section patrons: Comprised of individuals who could conceivably use the library for their income or employment needs, including people who are currently employed for wages (part- or full-time), entrepreneurs, and unemployed job seekers.
• Other patrons^6^ : Patrons not included in the above two groups, e.g. the retired, unemployed not seeking for a job, homemakers, and those who refused to state their primary occupation.

Other evaluation respondents included educators, key informants, and staff. Educators consisted of administrators, teachers, and school librarians at secondary schools within each regional library catchment area who may or may not use the libraries; target key informants were primarily government officials at the national and regional levels, and people associated with librarian professional development in the country; staff included the chief librarian, library section heads, and IT staff at each regional library (Coward et al., 2019).

### 4.2 Evaluation instruments

For phase two, seven types of data collection methods were used: (1) surveys (patrons), (2) panel studies (learner patrons, business section patrons), (3) interviews (key informants, staff), (4) focus group discussions (learner patrons, business section patrons, educators, general community patrons), (5) secondary data analysis (administrative reports, electronic system-generated data), (6) media analysis, and (7) observations.

Each data collection method reflects one of the three “perspectives”: patron, library, and external. The reason for taking this approach was to adequately understand the perspectives of each group before incorporating the views of other groups.

• The patron perspective: represents the experience of people who use the library, as informed by the *patron surveys, panel studies, and focus group discussions (FGDs)*.
• The library perspective: represents the views of individuals who work at or oversee the libraries in some capacity. Methods included *staff interviews and key informant interviews*.
• The external perspective: provides a perspective removed from the views of research subjects. Methods included *direct observations, secondary data analysis, and media analysis*.

For the interim evaluation report, within the patron perspective, survey data was to be complemented (or challenged) by panel interview data, then contextualized with FGD data. For the library perspective, summarized staff interview data and key informant interview data were to be integrated. For the external perspective, data sources were to be combined where applicable. The intention was to carefully integrate the three perspectives with an eye toward comparing and contrasting views and finding gaps in each perspective as well as providing a sequence for integrating data (more details on analytical decisions and process of integrating across the three perspectives are explained in Section 4.3).

The findings from patron surveys, panel studies, and secondary data were expected to quantify what was illuminated in qualitative findings. Qualitative methods (FGDs, staff and key informant interviews) aimed to illuminate patrons’ or service providers’ perceptions of library performance, outcomes from regional library use, and status of operations (Coward et al., 2014).

We will now look into each data instrument in further detail to show how they were designed. Please refer to Table 2 for the actual number of participants.

**Table 2:** Data collection activities.

Perspective	Approach for data collection and analysis	Data collections		No. of respondents or incidences
Patron	Quantitative	Patron surveys	Youth learners	144
			Adult learners and students	140
			Business section patrons	139
			Other patrons^c^	27
	Quantitative (Partly qualitative^a^)	Panel studies	Learners	60
			Business section patrons	60
	Qualitative	FGDs^b^	Learners^d^	12
			Business section patrons	10
			Educators	16
			General community patrons	7
Library	Qualitative	Interviews	Key informants	9
			Staff	15
External	Qualitative	Observations		6 (incidences)

a Included short responses to open-ended questions during interviews.

b Recruiting focus group discussants was very challenging. There were cases where the contacted potential discussants initially agreed to participate over the phone but did not show up on the arranged date. As a result, some focus groups were held in small numbers, not large enough to allow for group discussions.

c “Other patrons” were not included in the interim evaluation report analyses as a discrete population of interest; the sample size was too small to provide meaningful claims (Coward et al., 2019).

d For the learner FGDs, only one participant was in age group 20–24, and rest of the participants were in age group 15–19 (i.e. "youth learners").

#### 4.2.1 Patron surveys

The primary purpose of the patron survey was to support understanding of who uses libraries and in what ways. The survey sample was stratified to achieve sufficient sample sizes of secondary school learners, business section patrons^7^, and general community members. 8 Patron sub-groups included: job-seekers, entrepreneurs, waged employees, learners in secondary school, adult learners and students, and other general users. The survey was designed to be administered twice, once in 2017 and once in 2018, and provide cross-sectional data of general trends in usage patterns among target populations.

#### 4.2.2 Patron panel studies

The panel studies were designed to focus on two groups of patrons: secondary school learners and business section patrons (individuals who use the libraries for business, employment, or income-related purposes). The primary objective was to monitor their library use and report on patron outcomes. Between 2017 and 2018, it was planned to have an initial face-to-face semi-structured interview, three follow-up telephone surveys, and a final interview, in three-month intervals. The participants were to be randomly drawn from the pool of learners and business section patrons who completed a patron survey, using their unique IDs.^9^ In contrast to other patron perspective methods, panel studies could generate longitudinal data, tracking changes in patrons’ experience over a period of time, including individuals who stopped using the library. Moreover, by recruiting participants from those who answered patron surveys, panel studies were designed to examine context of the usage patterns illuminated in the survey findings.

#### 4.2.3 Focus group discussions (FGDs)

The intention of FGDs was to assess aspects of effectiveness, satisfaction, and user needs. Four groups would participate in FGDs at each regional library location: secondary school learner patrons, business section patrons, educators (may or may not be patrons), and general community patrons. A sample of discussants was to be drawn from either patron survey participants or snowball sampling. Educators were to be identified and selected from multiple schools in each regional library catchment area. FGDs help depict contextual detail behind the trends and changes not captured by the use of quantitative methods. Moreover, in comparison to patron surveys or panel studies, FGDs can elicit group dynamics. With the considerate role of facilitator, participants may express and exchange diverging views on a topic. Moreover, what could have been neglected as a minor issue during individual interviews can be revealed as a significant topic in a group (Wildemuth, 2009 cited from Williamson, 2013).

#### 4.2.4 Key informant and staff interviews

The main objective of the key informant and staff interviews was to explore factors facilitating or inhibiting the libraries’ ability to achieve service, population, and outcome targets, and also to understand operational aspects of the libraries. It was planned to have two rounds of interviews during the data collection period. From the library perspective, exploring *both* key informants’ and staff’s views is necessary as they complement each other. The former would inform how the libraries have operated in pursuant of their overarching objectives and how they are managed in cooperation with broader stakeholders such as local government. The latter would reveal day-to-day operations at the library, being able to fill in the gap between supervisory vision (from key informants) and users’ claims and challenges (from patrons).

#### 4.2.5 Secondary data analysis

Secondary data analysis was designed to gather monitoring data collected by NLAS to learn about service provision and usage rates. The main sources were expected to be: NLAS regional library usage reports (information on the usage on a quarterly basis); staffing updates (data gathered on the number of filled and vacant positions from each regional library on a monthly basis); data collected through a centralized integrated library management system (ILMS) (circulation and membership data); mobile library unit (MLU) service delivery and usage records (if available); and community needs assessment (CNA) data and reports.

#### 4.2.6 Media analysis

Media analysis was intended to examine the external perception of the regional libraries and their accomplishments. The target data would be from media sources, including publications on significant events and pronouncements related to the libraries. Articles were to be sourced from online media outlets in Namibia (e.g. national or local newspapers and magazines). Offline media sources could also be included, if readily accessible. Social media posts were excluded.^10^

#### 4.2.7 Observations

There were dual objectives for the observations: to examine if expected library activities were taking place and exploring how patrons utilize the libraries with a goal-free evaluation lens. It was planned to conduct observations multiple times at each regional library in 2017 and 2018 on “typical” days around the dates when other types of data collection took place. Observations would be conducted during regular opening hours, with each observation between one to four hours in length (depending on the degree and/or types of activities taking place), divided into morning and afternoon visits. Areas of observations included *visitors* (number of visitors, male or female dominance, interactions with other visitors, library materials used), *staff* (visibility and activities, interaction with patrons), *IT and equipment* (WiFi speed, computers, photocopiers/printers, lights/electrical), and *setting* (volume/noise, displays and signage, orderliness/cleanliness) in different sections of the library (i.e. general library and grounds environment, business section, study space, computer areas, and children’s section).^11^ Moreover, if observable, activities of mobile libraries and programs or classes were also planned to be noted.^12^

### 4.3 Analysis

The overarching analysis plan was to integrate and triangulate the three perspectives (explained in Section 4.2). Operationalizing this required determining which data sources could be used to answer each evaluation question (EQ). EQ2 (service provision and operations) was examined with data from all three perspectives—patron, library, and external. Addressing EQ3 (use), on the other hand, relied more on data from the patron perspective (as noted in Section 6.2.4, we encountered some limitations while combining data across the three perspectives for EQ3).

Optimal integration among the three perspectives was possible when there was close alignment across methods around specific topics within an evaluation question. For example, analysis of library services examined their usage (patron perspective) and provision (library perspective); e.g. findings from FGDs (patron perspective) were integrated with library perspective findings to illustrate how patrons’ use was affected by operating hours.

Data from the external perspective was used for specific purposes in the interim evaluation report. Secondary data was useful for examining EQ2 and EQ3. Observation data sometimes helped corroborate views that were not substantiated elsewhere (e.g. the views of one or a few focus group participants), and yet could have been integrated with other data sources more methodically. Media analysis is being saved for the final report since by design, it is primarily intended to shed light on EQ6 (promotion of the libraries) and EQ7 (influence of the libraries beyond the walls), which were not the primary interest of the interim evaluation report (EQ2 and EQ3).

With the above decisions, the following steps were taken to analyze and integrate data.

1 *Identify major themes based on the evaluation questions of primary interest for the interim evaluation analysis and report.* Based on evaluation questions of primary interest for the interim evaluation analysis and report (EQ2 on types of resources and programming the libraries provided and EQ3 on use), major themes to be examined were identified. The major themes helped inform qualitative data coding and report structure.
2 *Analyze data for each data source separately*. Patron surveys were analyzed in R to produce descriptive statistics. Others such as the FGDs, panel interviews (short responses), staff and key informant interviews, and observations were analyzed using coding schemes specific to each method, using Dedoose, Word, and Excel.
3 *Integrate data analyses across methods*. For the themes pertaining to EQ3 (library use: by whom, why, and how the libraries are used, satisfaction of users), patron perspective findings from quantitative methods (mainly patron survey, and parts of panel studies) led the analysis and were triangulated and/or contextualized with qualitative FGD data where applicable. For aspects related to EQ2 (types of resources and programming the libraries provided), library, patron, and external perspectives’ findings were integrated where applicable. According to Fetters, Curry, and Creswell’s (2013, pp. 2142) classification of levels and methods of integration in mixed-methods research, the evaluation’s integration took place at “interpretation and reporting level” and used “weaving approach” which involves “writing both qualitative and quantitative findings together on a theme-by-theme” basis.
4 *Identify patterns within each section of the interim evaluation report*. After all of the data had been organized and analyzed, a broader look was taken to summarize and capture key findings for each section of the interim evaluation report.
5 *Consult with stakeholders*. The evaluation team held a one-day workshop in Namibia with leaders of the regional libraries and Ministry of Education, Arts and Culture (MoEAC) to discuss their interpretation of a selection of data using data placemats (Pankaj & Emery, 2016) and obtain their input on preliminary evaluation findings. The evaluation team and members of the local data collection firm took notes, documenting the discussion, which informed some minor changes to the first draft of the evaluation report. Changes were documented in an appendix of the report.

## 5 Results

Table 2 shows the actual number of participants in patron surveys, panel studies, FGDs, and interviews.

This section presents four discrete areas of findings to illustrate how we employed a mixed-methods approach in order to (1) assess multi-faceted library performance (in terms of use and/or operation) and (2) achieve information complementarity. Regarding the latter, two areas of findings (5.1 Information and communication technologies (ICTs) and 5.2. Facilities) illustrate how data was integrated from two of the three perspectives; the other two (5.3. Collections and 5.4 Staffing) integrate all three perspectives.

### 5.1 Information and communication technologies (ICTs)

In terms of *revealing multi-faceted library use*, patron perspective methods—surveys, panel interviews, and FGDs—revealed how ICT resources and services were being used. Patron surveys also revealed high popularity of ICT services and resources, such as the WiFi connection, printers, photocopying machines, and scanners. FGDs provided a more contextual illustration of how the ICT facilities in the libraries were benefiting the patrons. For example, business section patrons’ responses portrayed how the entire process of job searching—browsing work descriptions, typing CVs, scanning relevant documents, applying online, receiving feedback—can be done through free or affordable ICT services at the libraries. Moreover, when it was asked how the libraries supported patrons in achieving their goals, many focus group discussants mentioned that the Internet was a source of help—for example, they could find information (e.g. find books or study materials online) or save money in applying for jobs (compared to using Internet café to send applications). Yet, the data from the patron perspective were also critical in revealing the challenges faced amidst the growing demand. To illustrate, patron survey response illuminated a noticeable number of patrons possessing concerns about computer availability (around one-seventh of learners/students and nearly one-tenth of business section patrons). FGDs provided explanations, that the patrons perceived time allocated for computers (up to 60 min) is too short to complete what they wish to do.

Just assessing library ICTs from the patron perspective would have left a major gap in answering *why* the challenges occur and *if* adequate responses have been in place. Contributing to the *information complementarity*, staff and key informant interviews—representing the library perspective—filled in the missing pieces of the puzzle by providing reasons behind the issues and the managements’ efforts to resolve the issues. Regarding computer use rules, staff generally felt the need to control the computer use so that a range of patrons could have a chance to use them. Mindful of the complaints, staff allowed some flexibility to those with more time-consuming work (e.g. doing homework) or designated several computers in certain library sections to be exceptions to the rules. In comparison, library perspective findings also informed that in other instances, staff responded to high computer demand (and resulting slowness of the Internet) by restricting computer use by school children from Mondays to Thursdays, allowing use only for certain purposes (completing homework or doing research). Increasing the number of computers was not perceived to be a feasible option under budget constraints, but IT staff perceived the maintenance of computers as a priority task, as was revealed during the interviews.

The evaluators suggested that the libraries revisit computer usage policies, for example by dedicating some computers to schoolwork use—which might require longer time to complete, compared to other uses such as for entertainment—at certain times during the day.

### 5.2 Facilities

First, in terms of *assessing various aspects of library performance*, the methods pertaining to the patron perspective illuminated different aspects of the facilities’ setting and status. Responses to patron surveys, panel interviews, and FGDs showed that a noticeable number of patrons perceived the library as a safe and welcoming place conducive to carrying out tasks or relaxing. For example, when patron survey respondents were asked to identify the top three elements of the library that they are most satisfied with, around one-fifth of learners and students and nearly a quarter of business section patrons mentioned the availability of seats. Also, around one-fifth and one-eighth of survey respondents included safety and ability to relax respectively, as one of the top three satisfactory aspects. However, the same methods also helped to identify areas of user concerns. One area was noise level. Around one-third of patron survey respondents included noise as one of the three most dissatisfactory aspects of the library. FGDs provided a more detailed picture of the cause as it revealed that the ability to concentrate was negatively affected largely by the sound of children. Satisfaction on safety was also challenged as some focus group discussants described that they felt unsafe due to uncleared bushes around the library building and the absence of a fence, which makes people vulnerable to robbery when they are using WiFi outside the library after its operating hours.

Second, in terms of providing *complementary information* to the patron perspective, the library perspective—staff and key informant interviews—were integrated to provide information on library performance from the service provider end, which was not readily revealed from the user end. The findings informed that the status of air conditioners, roofs, power generators, solar power equipment, and water pressure differed across the three venues and that some were experiencing difficulties in maintenance and repair for a variety of reasons, including limited town infrastructure (e.g. water lines), the challenges of working with contractors (e.g. incompatible invoicing procedures, proprietary systems), and limited financial resources to fund facilities upkeep.

The evaluators suggested that the libraries should find ways to keep noise within the children’s section—such as by keeping the door to children’s section closed or placing installations to reduce sounds—so that it would not disturb patrons in other sections. Moreover, the libraries should bring in a professional to fix the generators, solar power and other equipment sooner than later, considering that the long-term costs incurred as a result of not fixing them would be greater compared to the cost today.

### 5.3 Collections

In examining collections, integrating all three types of perspectives (i.e., patron, library, and external) was particularly valuable in showing and assessing the gap between how patrons valued the collections and their actual utilization of resources—contributing to *information complementarity*. First, from the patron perspective, panel interviews and FGDs illustrated that patrons regard collections as an important library resource. During panel interviews, around one-third of learners and nearly one-fifth of business section patrons responded that they valued books the most in the regional libraries. FGDs provided more detailed reasons behind the significance of collections in the libraries—the venue offered collections that are expensive to buy (e.g. exam preparation or study booklets) or not often found in schools (e.g. storybooks, picture books, reference materials).

However, the findings pertaining to significance of collections were challenged by the external perspective—secondary data analysis of administrative data revealed that the number of items borrowed from the libraries (during the year of 2016) marked significantly lower than the annual baseline target established by the MoEAC and MCC before the libraries opened. The library perspective suggested that the low level of items borrowed could have been influenced by a shortage of books available. Previously, phase 1 key informant interviews informed that a failed book order due to difficulties in procurement resulted in significant gaps in the initial collection—some key informants then expressed concerns that the shortage of books might hinder achieving library’s goals (Sey & Fellows, 2016). The results of shortage in supply of collection were partly evidenced in phase 2 findings which addressed the patron perspective. For example, FGDs revealed patrons’ demand for certain types of books to be added to the libraries’ collections. Across the three venues, items frequently mentioned included: school textbooks, exam preparation books or booklets, fiction (including poetry books or storybooks), and books in languages other than English.

The evaluators suggested that there should be a long-term plan for library collection development. Considering the budget constraints, it might be more feasible for the libraries to develop a five-year or ten-year plan for collection development, rather than planning to acquire necessary collections at once. Partnerships with organizations in and outside Namibia can help the regional libraries to receive books, videos, DVDs, and CDs.

### 5.4 Staffing

Staffing was another area where the use of multiple data collection methods and integration of different perspectives contributed to *information complementarity*. Overall, they together informed the percentage of positions filled (from administrative data; external perspective), views and issues on hiring, retaining, and training staff (from staff and key informant interviews; library perspective), and perspectives on staff helpfulness and attitudes (from patron survey, FGDs, and panel interviews; patron perspective).

First, administrative data showed that there is a shortage of staff in the libraries. For each regional library, around 34–36 positions were approved. However, as of August 2017, a quarter of positions were not filled in two locations, and about a half of positions were vacant in one location.

The library perspective provided an explanation for the shortage of staff in terms of high turnover and budget constraints. Staff interviewees mentioned that turnover occurred because staff sought and left for other opportunities. Staff and key informants mentioned possible reasons for turnover: people being better paid at—and thus attracted to—private sector or other libraries; pursue education; and preference to work in urban areas. Moreover, in 2017, government budget cuts led to a freeze in hiring staff in the regional libraries which made it difficult to hire crucial positions such as chief librarian (one location) and IT section head (two locations). Staff and key informant participants also shared how the libraries were coping with the challenges by e.g. allowing flexibility of staff roles to fill in the gaps, and loosening qualification requirements for library assistants and giving them on-the-job training. Staff interviews also informed that there is a slight gap between the trainings that were offered by the regional libraries under the budget constraints and the trainings the staff would like to receive more. Some training opportunities were available in-house (e.g. staff-led trainings on Excel or librarianship) and externally (a few staff participating in trainings provided by outside organizations) and the regional libraries supported several staff in pursuing university degrees in librarianship. Yet, staff wanted more professional development closely associated with the sections they work in, e.g. childhood development (children’s section) and knowledge in business plans and marketing (business section).

In this setting, the patron perspective on staff helpfulness and attitudes reflected patrons’ level of satisfaction with staff services under the challenges and constraints explained above. First, patron views were divided on helpfulness of staff. When patron survey asked the three most satisfactory library features, helpfulness of staff was selected by 19 % of learners/students and 26 % of business section patrons. On the other hand, it was also mentioned as one of the three least satisfactory library features by 14 % of both patron groups. Supporting the latter finding, several FGD participants mentioned occasional difficulties in seeking available staff for assistance or finding skilled staff to help them. This was in contrast to staff responses in two locations as they mentioned they were becoming adapted to operate with around 75 % of positions. Second, views on staff attitudes were also mixed. During the panel interviews and FGDs, some patrons complimented staff’s friendliness, willingness to help, and strong commitment to work, whereas others criticized staff’s impoliteness and hostility toward patrons seen in some occasions.

Overall, the patron perspective informs us that there is room for improvement in staff services. Evaluator suggestions included seeking more affordable ways to provide staff training (e.g. free online courses), and promoting and rewarding skills and passion for designing new programs, helping patrons, teaching ICTs, etc.

## 6 Discussion and conclusion

### 6.1 Mixed methods in Namibia regional library evaluation: Potentials revisited

In Section 2, we explored key aspects of library evaluation and what mixed-methods approaches can potentially offer. To recall, we saw that scholars have examined areas such as economy, efficiency, effectiveness, productivity, service quality, use, and user satisfaction in library performance evaluation in an effort to conceptualize the connections among input, process, output, outcomes, and impact. We argued that the use of mixed-methods approaches can support the assessment of multi-faceted library performance. It is beyond the scope of this paper to present evaluation findings of all the above aspects. Yet, in the results section (Section 5), we saw how the application of a mixed-methods approach could reveal aspects of effectiveness (e.g. how business section patrons utilize ICTs throughout the job search and application process in alignment with libraries’ objectives), use (e.g. number of items borrowed), and user satisfaction (e.g. why some patrons are concerned with noise level and safety) in areas of ICTs, facilities, collections, and staffing.

In capturing the above aspects, the strength of using mixed methods to assess library performance was demonstrated mainly twofold. First, a mixed-methods approach allows an evaluator to position oneself in the intersection of two dimensions—trends/patterns/themes on one hand and context/rationale/implications on the other. For example, the patron survey (a quantitative method) illuminated the trend or level of usage of ICT services and resources, whereas FGDs (a qualitative method) provided contextual information of how the ICT facilities in the libraries were benefiting the patrons. Second, the approach enables exploration of the intersection from multiple perspectives—in our case, patron, library, and external perspectives. This supports previous studies’ arguments that mixed-methods approaches can contribute to “information complementarity,” “contextualised analysis of …impacts,” and reflection of a “diversity” of actors and viewpoints (da Costa et al., 2013, pp. 7–8). Moreover, it shows the operationalizability of library evaluation model employing the library perspective and/or the patron perspective (as suggested by Nicholson, 2004) through mixed methods.

The potential of mixed-method approaches in library evaluation can be realized through careful consideration on the strengths of each data collection method in terms of the type of information it can illuminate (e.g. the choice between surveys and interviews) and the dynamics it can generate in revealing the targeted perspective (e.g. the choice between patron FGDs and patron interviews). The dynamics can be shaped by factors such as power (im)balances (e.g. between evaluators and respondents, or between respondents in a group setting) and the level of knowledge of or experience with the libraries. Considerations of the dynamics and subsequent choice of methods will ultimately influence the depth and breadth of information gathered from a certain perspective. For instance, because of their age, secondary school learners might feel more intimidated compared to staff during a 1:1 interview with an evaluator. Also, staff might be more knowledgeable on how the libraries are run and what types of services are offered, compared to patrons who visit the libraries only occasionally. Therefore, it can be a reasonable choice to conduct FGDs with patrons to gather in-depth qualitative data, shifting the role of evaluator as a facilitator and eliciting nuanced impressions and opinions.

### 6.2 Limitations, challenges, and lessons learned

Despite the strengths in examining various aspects of library performance from multiple viewpoints, the evaluation is subject to some limitations. Moreover, during the evaluation and analysis process the team was faced with notable challenges. From the limitations and challenges, we derive our lessons learned. In particular, discussions presented in Sections 6.2.3–6.2.6 contribute to the practice of mixed-methods evaluation by suggesting areas for consideration for evaluators when using mixed methods.

#### 6.2.1 Assessing significance of the evaluation results

First, it is difficult to determine the extent to which the performance of the regional libraries was unique and particularly significant in the country compared to other libraries or ICT facilities. Considering that the regional libraries were established with the symbolic meaning to stimulate reading, learning, entrepreneurship, and community involvement throughout Namibia, examination of the regional libraries’ performance vis-a-vis other facilities serving a similar purpose might be important in the longer run.

#### 6.2.2 This study’s approach for statistical analysis

For patron survey analysis, we used descriptive statistical analysis of the stratified random sample data to summarize the responses. It was out of scope of this evaluation to establish a statistical relationship between variables such as regional library resource use and user outcomes based on inferential statistics, through e.g. correlation, hypothesis testing, etc. However, within the scope of the evaluation project, to examine possible associations between regional library resource use and user outcomes, we did have some questions in patron survey, panel interview, and FGD where the responses can shed light on outcomes resulted from the library use. It was out of scope of this evaluation project to use randomized controlled trial (RCT).

#### 6.2.3 Understanding cross-cutting themes of interest

In terms of analysis, greater integration of data across different perspectives and methods could have taken place to facilitate the understanding of cross-cutting themes of interest. For example, the team tried to capture differences between user gender groups and differences among the three library locations. At the time of analysis for the interim evaluation report, these differences were examined where applicable, but the analysis was subordinated under respective report sections (linked to different aspects of EQ2 and EQ3) and under patron types (i.e. learners and business people). This weakened the overall narrative of a theme to be examined, making it somewhat challenging to capture the full picture pertaining to gender or location differences. The narrative could have improved if more time was devoted to the examination of these themes.

#### 6.2.4 Integrating perspectives to address aspects of the evaluation questions

In this study, more profound integration could have taken place across different methods. For example, data from staff interviews could have been further integrated along with data from focus groups and patron surveys to show staff perspectives on patrons’ activities. In reality, this was a challenge—for instance, the staff interview questions were designed and responses were coded to describe usage in a narrower way than the patron perspective methods. Specifically, staff participants were asked about the use by target groups the library hoped to serve rather than asking about who was actually using various library services. As a result, the library perspective mostly shed light on usage in regard to the success of service provision, while patron and external perspectives directly addressed actual usage patterns (i.e. through patron surveys, FGDs, panel interviews, and administrative data). What this illuminated during data analysis were the limitations and difficulties in balancing between (1) focusing only on the primary aspects of evaluation questions that a particular method was originally designed to capture versus (2) integrating all data across different methods in all possible instances. In the case for interim evaluation analysis and reporting, primary emphasis was placed on the former, while the latter became secondary. An alternative set of qualitative coding and analysis strategies that more closely balanced both of the above aspects could have facilitated greater integration across methods—albeit, with the additional time required for finely-detailed analyses. We plan to use this reflection to adjust how we approach the analysis for the final report.

#### 6.2.5 Importance of planning for analysis

Since the performance evaluation involved gathering findings from seven distinct methods addressing multiple themes corresponding to evaluation questions, collecting and analyzing the data and reporting the findings were time consuming processes. We learned that, if not designing a mixed-methods approach so each round of data collection shapes the next round, it is important to connect the evaluation questions, methods, and analysis approach from the beginning—ultimately to avoid any redundant or duplicative data being produced.

In planning for analysis, we also learned the challenges of adhering to a data analysis strategy timeline. Ideally, results from each method would become available concurrently, but the realities of various schedules (associated with data collection and of researchers) and other practical considerations led to a more staggered timeline, requiring a flexible sequence of steps for data analysis and integration around each evaluation question.

#### 6.2.6 Importance of internal and external collaboration

Internally within the research team, building and maintaining collaborative work practices among the research team members are essential during analysis, especially for an evaluation at this scale. As analysis tasks were divided among the evaluators by method, active communication within the team was crucial for effective and efficient processes in integrating and writing of findings and analyses. The challenge was foreseen—“Challenges to integration…arise from paradigmatic differences in philosophical orientation and disciplinary traditions associated with different methods, personal predispositions and preferences of researchers, the training and skills of researchers, and conflicts within mixed-background teams” (Bazeley, 2018, pp. 11). To overcome the challenge, the evaluation team held both plenary meetings (e.g. weekly team meetings to regularly check on progress, all-day analysis workshops involving all evaluators) and 1:1 meetings (e.g. between evaluators involved in qualitative or quantitative analysis).

Externally with the participants, we faced the task of facilitating practical utilization of the evaluation results in Namibia. To achieve this, preliminary evaluation findings for the interim evaluation report were shared with in-country stakeholders (primarily regional library leaders and related government officials) in Spring 2018. Doing so helped to build trust with the stakeholders, increase the report’s accuracy, and better interpret ambiguous findings. Additionally, we learned how sharing preliminary findings can help stimulate discussions on systemic challenges in operating the libraries and provide a chance for participants to share ideas for solutions.

## Funding

This work was funded by the Millennium Challenge Corporation (MCC), U.S. The MCC had no involvement in analyzing the data nor writing of the article. The views and opinions expressed herein are those of the authors and do not necessarily represent those of MCC or any other U.S. Government entity.

MCC makes all of the reports from completed independent evaluations publicly available via the agency's Evaluation Catalogue. See here for details: https://www.mcc.gov/our-impact/independent-evaluations.

## CRediT authorship contribution statement

**Moonjung Yim:** Formal analysis, Writing - original draft, Writing - review & editing. **Michelle Fellows:** Methodology, Formal analysis, Investigation, Writing - original draft, Writing - review & editing, Project administration. **Chris Coward:** Conceptualization, Writing - original draft, Writing - review & editing, Supervision, Project administration, Funding acquisition.

## Declaration of Competing Interest

The authors declare that they have no competing interests.

## Footnotes

2 Evaluation questions addressed in this study—EQ2 (What types of resources and programming are the libraries providing?) and EQ3 (Who uses the libraries and what do they do?)—concern the areas of resources provided, usage, and operations.

3 The areas align with the purpose of the evaluation which is about understanding if the project was 'effective' and why or why not. One could argue that use, service quality, and user satisfaction are all elements of 'effectiveness'—they help explain why the libraries may or may not be effective at e.g. helping communities access information and develop skills.

4 Specifically the interim evaluation report, which aligns with the scope of this article.

7 Business section patrons indicates business section target patrons who are job-seekers, entrepreneurs, and waged employees.

8 We spoke with individuals age 15 and above.

9 In practice, due to challenges in recruiting sufficient numbers of patrons at some of the regional libraries, “all male and female secondary school learner patrons at the Oshana [regional library] and female secondary school learner patrons at the Ohangwena [regional library] were invited for panel interviews.” (Coward et al., 2019, p.29).

10 The purpose of the media analysis was to answer EQ7 (the influence of the libraries beyond their walls). To address EQ7, we wanted a perspective from sources that were independent (not library users or staff) and far-reaching (regional or national reach). Compared to newspapers, we expected social media would be used mostly by the libraries and their patrons, and we were already capturing their perspectives through other methods.

11 Specific areas of observation differed according to the resources available in, or structure of, each section.

12 In 2017, two people carried out the observations.

## Footnotes

5 In Namibia, those who attend secondary school are typically referred to as “learners,” while those who attend a tertiary educational institution such as university are called “students.”

6 “Other patrons” were not included in the interim evaluation report analyses as a discrete population of interest; the sample size was too small to provide meaningful claims (Coward et al., 2019).
